# Emerging Insights into the Metabolic Alterations in Aging Using Metabolomics

**DOI:** 10.3390/metabo9120301

**Published:** 2019-12-13

**Authors:** Sarika Srivastava

**Affiliations:** Fralin Biomedical Research Institute at Virginia Tech Carilion, 2 Riverside Circle, Roanoke, VA 24016, USA; Sarika_Srivastava@vtc.vt.edu; Tel.: +1-540-526-2047

**Keywords:** metabolomics, aging, metabolites, biomarker, metabolism, MS, NMR, model organisms, human longitudinal studies

## Abstract

Metabolomics is the latest ‘omics’ technology and systems biology science that allows for comprehensive profiling of small-molecule metabolites in biological systems at a specific time and condition. Metabolites are cellular intermediate products of metabolic reactions, which reflect the ultimate response to genomic, transcriptomic, proteomic, or environmental changes in a biological system. Aging is a complex biological process that is characterized by a gradual and progressive decline in molecular, cellular, tissue, organ, and organismal functions, and it is influenced by a combination of genetic, environmental, diet, and lifestyle factors. The precise biological mechanisms of aging remain unknown. Metabolomics has emerged as a powerful tool to characterize the organism phenotypes, identify altered metabolites, pathways, novel biomarkers in aging and disease, and offers wide clinical applications. Here, I will provide a comprehensive overview of our current knowledge on metabolomics led studies in aging with particular emphasis on studies leading to biomarker discovery. Based on the data obtained from model organisms and humans, it is evident that metabolites associated with amino acids, lipids, carbohydrate, and redox metabolism may serve as biomarkers of aging and/or longevity. Current challenges and key questions that should be addressed in the future to advance our understanding of the biological mechanisms of aging are discussed.

## 1. Introduction

**Figure 1:**
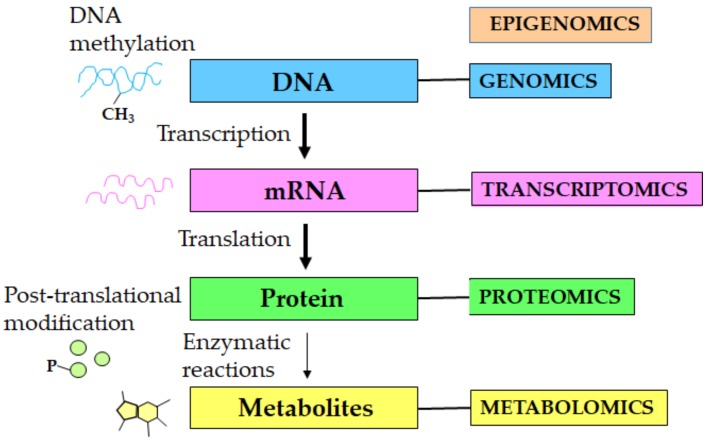
Systems biology approaches in aging research. Aging is associated with changes at the molecular, cellular, tissue, and organismal levels. The systems biology ‘omics’ technologies i.e., genomics, transcriptomics, proteomics, and metabolomics enable high-throughput quantitative profiling of molecules in biological systems to reveal global changes that are associated with aging. Integration of the multi-layered omics data is highly critical for a complete understanding of the biological mechanisms of aging. Notably, unlike the DNA, mRNA, and proteins, the metabolites are not directly involved in the “central dogma” of information flow.

Metabolomics is an emerging ‘omics’ tool and the systems biology science that involves systematic identification and quantification of a complete set of metabolites or low-molecular-weight intermediates (i.e., molecular weight < 1.5 kDa) in a biological system (i.e., cell, tissue, organ, biological fluids, or organism) in response to the pathophysiological stimuli, genetic modification, or environmental factors [1,2,3,4]. Metabolites (e.g., amino acids, sugars, nucleotides, lipids, organic acids) perform diverse functions in a biological system (e.g., energy production, macromolecular synthesis, signaling) and they provide a functional signature of the phenotype in response to the information that is obtained from genes, transcripts, and proteins. The total number of primary metabolites in biological systems is currently debatable, although it was reported that yeast contains a few hundred metabolites [5], humans contain thousands of metabolites [6,7], whereas plants contain hundreds of thousands of metabolites [8,9]. The ‘metabolome’ represents the complete complement of small-molecule metabolites in a biological system [10,11], which varies in response to the genomic, transcriptomic, proteomic, or environmental alterations, and is based on the physiological, pathological, or developmental state of the biological system [12,13,14,15]. The metabolome is not directly involved in the “central dogma” information flow, unlike the genome, transcriptome, and proteome (Figure 1), and indeed the human metabolome is much smaller than the proteome, which makes it relatively easier to characterize using the high-content and high-throughput system biology approaches [16].

In the past 15 years, two analytical platforms have predominantly emerged for performing metabolomics studies i.e., mass spectrometry (MS) and nuclear magnetic resonance (NMR). However, analytical techniques have their own advantages and disadvantages, and no single analytical platform has the ability to capture the entire metabolome [17,18,19,20,21]. Notably, the use of combined analytical techniques complement each other and broaden the level of metabolites coverage and sample types in the metabolomics experiments [19,22,23,24,25].

Two approaches are commonly used for metabolite profiling i.e., the targeted or “bottom-up” and an untargeted or “top-down” approach. A specific hypothesis drives targeted metabolomics and it allows for the absolute quantitation of structurally known metabolites with high accuracy in the presence of internal standards [26,27,28]. Untargeted metabolomics or global metabolite profiling analysis measures all detectable metabolites and it serves as an unbiased, hypothesis generating, and discovery based approach that provides a wealth of new biological information and offers wide biomedical and clinical applications [17,28,29,30,31]. The success of metabolomics studies relies on samples collection, careful execution of experimental protocols, data acquisition, pre-processing and quality assurance, data analysis and integration, and the identification of metabolites [32,33,34,35]. The metabolomics studies generate a large amount of data, similar to other omics technologies, and the task of extracting meaningful information is complex and involves the use of Metabolomics Standards Initiative (MSI) [36]. Data analysis and integration remain challenging tasks for deducing regulatory relationships between biological factors and elucidate the complex physiological mechanisms of aging [33,37,38].

Several metabolite databases now exist that allow for the identification of metabolites during global metabolite profiling analysis, including the METLIN database, human metabolome database (HMDB), MassBank, LipidMaps, Madison-Qingdao metabolomics consortium database (MMCD), biological magnetic resonance data bank (BMRB), NMRShiftDB2 database, and the spectral database from the National Institute of Advanced Industrial Science and Technology (AIST) in Japan (https://sdbs.db.aist.go.jp) [6,7,39,40,41,42,43,44]. These repositories allow for a comparison of the tandem mass spectrometry (MS/MS) and/or NMR data that were obtained from biological samples to the data from model compounds catalogued in the databases, thereby enhancing the speed and cost-effectiveness of the global untargeted metabolomics studies. The HMDB (http://www.hmdb.ca) currently contains ~114,158 metabolites, and it is the standard metabolomic resource for human metabolic studies [6,7]. Notably, untargeted metabolome-wide association studies (MWAS) have emerged as a powerful tool for identifying the associations between metabolic phenotypes and disease risk, biomarker discovery, and in the investigation of complex biological systems [33,45,46,47].

**Figure 2:**
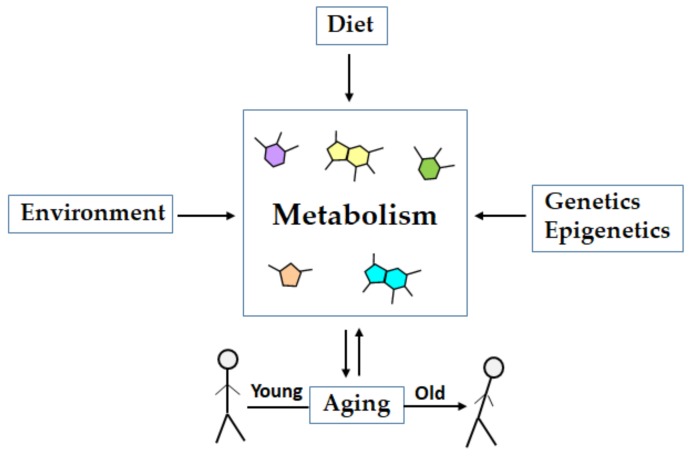
Metabolism integrates the effects of diet, environment, and genetics in aging. Aging is influenced by the alterations in genetics or epigenetics, diet and lifestyle factors, and environmental exposure. The phenotype of an organism is resultant of the interactions of the genotype with diet, lifestyle, and environmental factors. Metabolites represent the final fingerprint or snapshot of all molecular changes associated with a phenotype. Metabolic profiles thus integrate the effects of genetics, diet, and environment in aging.

Metabolomics measures both the downstream output of the genome and upstream input from the environment in a biological system, and thus allows for the identification of the endogenous (i.e., gene-derived) and exogenous (i.e., environmentally-derived) metabolites, and enables the exploration of the nexus of gene-environment interactions (Figure 2). [48,49]. Metabolomics studies performed in the brain, cerebrospinal fluid, or blood plasma have aided in unraveling the diversity and specificity of altered metabolites, as well as in discovering the potential biomarkers of aging and age-related diseases [13,50]. Thus, the omics technologies are at the forefront of aging research, and metabolomics led studies are emerging as a powerful tool for characterizing the metabolome of an organism that represents a myriad of chemical alterations and an integration of the response to genomic, transcriptomic, proteomic, or environmental alterations that occur with age. Currently, metabolomics have emerged as a robust and promising tool for identifying the links between genotypes and phenotypes, understanding pathological mechanisms, improving disease diagnosis, monitoring therapeutic outcomes, for biomarker discovery, drug discovery, drug toxicology, and in personalized medicine [2,17,31,45,51,52,53].

In this review, I will provide a comprehensive overview of our current understanding on the metabolomics led studies in aging from multiple model organisms, including worms, flies, and mice, as well as the large-scale human population based longitudinal studies that lead to the identification of age-associated metabolites changes and biomarker discovery. I will further discuss the current challenges and key questions in the field that remain to be addressed in the future for advancing our understanding of the complex biological mechanisms of aging. The ‘PubMed’ database search method was used to identify the articles with key search terms i.e., metabolomics, aging, biomarker, *C. elegans*, *Drosophila*, mice, and human.

## 2. Metabolomics of Aging

Aging is a natural biological phenomenon that is characterized by a gradual and progressive decline in physiological functions at multiple levels (i.e., molecular, organellar, cellular, tissue, and organismal), which result in an increased vulnerability to chronic diseases and death [54,55]. Genomic instability, telomere attrition, transcriptional and translational errors, and metabolites alterations are the primary hallmarks of the aging process [54,55,56,57,58,59,60]. The accumulation of irreparable macromolecular damage due to metabolically or environmentally generated free radicals, spontaneous errors in biochemical reactions, and the biologically programmed decline in the endocrine and immune system functions have been proposed as the main drivers of aging [61,62,63,64]. In addition, external factors, such as diet, physical activity, environment, and interaction with commensal and pathogenic microbiomes, can influence aging in various model organisms, including humans [65,66,67,68,69].

### 2.1. Biomarkers of Aging

A ‘biomarker’ or ‘biological marker’ was initially defined in 1998 by the National Institutes of Health Biomarker Definitions Working group as “a characteristic that is objectively measured and evaluated as an indicator of normal biological processes, pathogenic processes, or pharmacologic responses to a therapeutic intervention” [70]. Additionally, the International Programme on Chemical Safety (IPCS), which is a joint venture of the United Nations Environment Programme (UNEP), the International Labour Organization (ILO), and the World Health Organization (WHO) defined a biomarker as “any substance, structure, or process that can be measured in the body or its products and influence or predict the incidence of outcome or disease” [71]. Thus, a biomarker could be any measurable substance in an organism, the presence of which is indicative of a process, such as aging, disease, infection, or environmental exposure, and it can include both physical traits (e.g., blood pressure, pulse, or body temperature) and biological molecules (e.g., proteins, lipids, or amino acids) [72].

The biomarkers of aging mainly comprise the phenotypic and molecular biomarkers, which provide a measure of physiological age, examine the extent of healthy aging, and predict lifespan. The loss of strength, muscle tone, and skin elasticity, reduced visual acuity, flexibility, and cardiovascular stamina, as well as increased body fat are included as some examples of phenotypic biomarkers of aging, whereas examples of the molecular biomarkers of aging include the altered levels of hormones, lipids, glycosylated hemoglobin, glucose, amino acids, and cholesterol. These biomarkers are mainly used in the human population based studies to understand the physiological changes that occur with age, the process of aging, and the onset of age-related diseases [73,74,75,76].

### 2.2. Aging Studies in Model Organisms

Aging is associated with changes in systemic metabolism, including changes in the amino acids, lipids, sugar, and nucleotide metabolism. Targeted and untargeted metabolomics approaches have both been used to identify changes in diverse metabolites with aging in various model organisms, including worms [77], fruit flies [78,79], and mice [80,81]. For example, Fuchs et al. performed NMR based targeted metabolomics in three classes of long-lived worms and identified a common metabolic signature that is associated with metabolites alterations in the carbohydrate (i.e., elevated levels of trehalose sugar) and amino acids (i.e., elevated levels of branched-chain amino acids) metabolism, indicating that longevity pathways may regulate the same regions of metabolic network [77]. In another study, Avanesov et al. profiled >15,000 metabolites in flies that were subjected to control and life-span extending diets by untargeted liquid chromatography mass spectrometry (LC-MS) analysis, and found that aging is associated with increased metabolites diversity and low abundance molecules due to cumulative damage and gradual metabolome remodeling, and that lifespan extension in flies is associated with decreased metabolism and tolerance to damage [78]. A study by Hoffman et al. demonstrated that fly metabolome is highly sensitive to physiological state and it shows a dramatic variation in response to the age, sex, and genotype [79]. This study further identified several metabolic pathways that were enriched with metabolites changes, including the sugar and glycerophospholipid metabolism, amino acids, neurotransmitters, and carnitine shuttle [79]. Interestingly, a key tricarboxylic acid cycle (TCA) intermediate metabolite α-ketoglutarate was recently shown to extend lifespan in worms, flies, and mice, which suggested that longevity pathways might be evolutionarily conserved [82,83,84,85].

Methionine metabolism was also reported to be significantly altered with age, as demonstrated by a high-throughput targeted metabolomics study in flies revealing that S-adenosyl-homocysteine (SAH) metabolite accumulates with age, and that suppressing the age-dependent SAH accumulation in turn suppresses the H3K4 trimethylation (H3K4me3), thus mimicking methionine restriction and leading to the health span and lifespan extension in flies [86,87]. The effects of dietary restriction (DR) and age were also investigated in different tissues from flies, demonstrating that DR significantly alters the metabolome and impedes the age-related changes in the metabolome [88]. This study identified a network structure of metabolites that were altered by DR in flies, including the amino acids, nicotinamide adenine dinucleotide (NAD), and novel metabolic pathways, which shed mechanistic insights on the DR mediated protective effects on health span and lifespan in flies [88]. Interestingly, the fecal metabolomics approach recently examined the age-related differences in the metabolome of long-lived brown bats (i.e., a non-model mammalian organism), which demonstrated that 41 metabolites were altered between young and elderly bats with significant differences in metabolites that were associated with the tryptophan metabolism and incomplete protein digestion, indicating the importance of protein homeostasis and tryptophan metabolism pathways in longevity [89].

Tomas-Loba et al. reported a metabolomics signature that accurately predicts the biological age rather than chronological age in mice [80]. This study demonstrated that metabolomics score of telomerase deficient mice with a shortened lifespan predicted older ages than expected, whereas the metabolomics score of telomerase overexpressing mice corresponded to younger ages than expected. Interestingly, telomerase reactivation late in life significantly reverted the metabolic profile of old mice to that of younger mice, further indicating an anti-aging role of telomerase [80]. Another study by Houtkooper et al. compared the metabolic parameters of young and old mice and identified a distinguishing metabolic footprint of aging. This study demonstrated that glucose, fatty acid metabolism, and redox homeostasis pathways are altered with age, which leads to a decreased long-chain acylcarnitines and amino acids levels, as well as increased free fatty acid levels in the plasma of aged mice [81]. A study by Wijeyesekera et al. further characterized the plasma metabolic phenotype of three long-lived murine models and identified several potential candidate biomarkers of longevity, including metabolites that were involved in phosphatidylcholine metabolism, and specific differences in the metabolic signatures of all models were also identified, suggesting the multifactorial control of the aging process [90]. De Guzman et al. demonstrated that DR prevents the age-related alterations in specific metabolites involved in lipid metabolism, fatty acid metabolism, and bile acid synthesis pathways, while using global and targeted MS-based metabolomics approaches [91].

The metabolic basis of mammalian diversity and longevity were further quantified in different tissues (i.e., brain, heart, kidney, and liver) from 26 mammalian species by metabolomics profiling analysis and metabolite patterns that were characteristic of the organs, lineages, and species longevity were identified [92]. This study reported that the metabolites in brain diverged less than in other examined organs, and that metabolites patterns reflected the organ-specific functions and lineage-specific physiologies [92]. Moreover, the study identified the metabolites that correlate with species lifespan, and found that longevity in mammals is associated with low levels of polyunsaturated triacylglycerols, low tryptophan degradation products, low brain amino acids, high sphingomyelin levels, and a high urate to allantoin ratio [92]. Recently, the ‘energy metabolic drift’ phenomenon in the brain of aging mice was also reported, which demonstrated a significant metabolic imbalance in the core metabolites (i.e., NAD decline, increased AMP/ATP ratio, accumulation of purine/pyrimidine), alterations in the oxidative phosphorylation and nucleotide metabolism associated with brain aging, and suggested a loss of brain metabostasis (i.e., metabolite homeostasis) with an increasing age in mammals [93].

In summary, the metabolomics driven studies in aging from multiple model organisms demonstrated that aging is associated with increased metabolite diversity, changes in several metabolic pathways, and long-lived organisms exhibited a metabolic signature that is associated with shifts in carbohydrate, lipids, amino acids, and redox metabolism, which indicated that these metabolites can serve as biomarkers for aging and health span [77,78,79,80,81,94].

There are several gaps in our knowledge in the field and multiple avenues for future research exist, despite a significant progress in identifying metabolites changes that are associated with aging in model organisms. For example, it is important to determine which molecular or metabolites changes are the cause or consequence of aging in different organisms? Whether metabolites alterations exhibit tissue specificity with chronological age in different species? What are the potential mechanisms that regulate species lifespan? It is feasible to determine the differences in genomic, transcriptomic, proteomic, and metabolomic signatures among the short-lived and long-lived organisms, and identify key regulators of organismal lifespan, by using comparative omics approaches. Moreover, the high sensitivity omics approaches can allow a detailed investigation at multiple time-points, single-cell sequencing, and in vivo quantitative analysis using microfluidic platforms in different tissues and organisms, which could aid in identifying the potential cause-and-consequence relationships. Notably, the yeast metabolome database (YMDB) and the mouse multiple tissue metabolome database (MMMDB) were recently made available [95,96]; however, it is also a high priority to curate the metabolomes of all other model organisms used in aging research.

### 2.3. Aging Studies in Humans

A critical aspect of aging research is the profiling of molecular changes that occur with an increasing age in humans. The advent of high-throughput omics technologies (i.e., genomics, transcriptomics, proteomics, and metabolomics) have enabled a comprehensive molecular profiling of the biological systems to reveal global changes in molecular and metabolic pathways that occur with age [38]. The measurement of the changes in all components of the biological system, interactions between the individual components, and integration of the data from different experiments is vital for the complete understanding of biological mechanisms of aging at the systemic level [37,97].

A number of omics-based studies have revealed age-associated changes in the epigenome and gene expression during aging in humans and multiple animal models [98,99,100,101,102,103]. The phenotype of an organism is a result of the interactions of the genotype with diet, lifestyle factors, and environment (Figure 2), which exhibits variation with age and between individuals of the same chronological age [104]. The metabolomics studies identify the changes in metabolites levels that result from or contribute to a phenotype, or that are induced by treatments [105,106]. Thus the metabolites signature provides an overview of the metabolic state of a biological system and it is more sensitive than the transcriptome and proteome to the external factors that govern phenotypic changes in biological samples. Therefore, a comparison of the metabolic profiles allows for distinguishing between different physiological and individual stages, pathological states, and predicting the responses to pharmacological treatment [107,108].

Numerous metabolomics studies have explored age-related changes in metabolites levels and found that the metabolic profiles are strongly correlated with chronological age and/or longevity (Table 1) [56,59,94,109,110,111,112,113,114,115]. For example, Lawton et al. characterized the effect of age and sex on human plasma metabolome from a cohort of 269 individuals while using untargeted metabolomics, and found that more than 100 metabolites were significantly altered with age, whereas relatively few metabolites were altered with either sex or race [115]. Another study quantified changes in metabolites while using targeted metabolomics in fasting serum samples from adult humans in an age group of 32–81 years, and found that the metabolic profiles are age-dependent and they may reflect different aging processes [56]. A study by Menni et al. analyzed blood samples from a large twin population (i.e., 6055 individuals) while using untargeted metabolomics, and identified 22 metabolites that were significantly altered with age of which one particular metabolite i.e., C-glycosyl tryptophan (C-glyTrp) was found to strongly correlate with chronological age and lung function [111]. Moreover, the epigenome-wide association study identified three CpG sites that were associated with C-glyTrp levels [111]. Interestingly, the epigenetic DNA methylation pattern of a set of CpG islands i.e., the “epigenetic clock” has been shown to predict the biological age and survival in human longitudinal studies [116].

Collino et al. reported the metabolic signatures of longevity in a human aging cohort, and showed that serum tryptophan concentration is markedly reduced with increasing age, and that a unique alteration of specific glycerophospholipids and sphingolipids are associated with longevity phenotype, while using the combined NMR and targeted MS profiling approaches [94]. This study further revealed that the longevity process alters the structure and composition of human gut microbiota, such that the levels of phenylacetylglutamine (PAG) and p-cresol sulfate (PCS) are higher in the urine of centenarians, which indicates that a complex remodeling of lipids, amino acids, and gut microbiota metabolism mark the longevity in humans [94]. Another study investigated the urinary metabolic profiles with age of a Taiwanese and an American population, and reported that PAG and PCS metabolites are both positively correlated with age, suggesting an age-related association of the host-microbiome metabolism [112]. The authors further found that the levels of creatine and β-hydroxy-β-methylbutyrate (HMB) metabolites are negatively correlated with age in both populations, which indicates reduced muscle mass with age [112]. Cheng et al. performed metabolite profiling in a large human cohort by the LC-MS approach and found that polar compounds and lipid analytes were associated with longevity, including the citric acid cycle intermediates and the bile acid taurocholate [110]. The authors further found that none of the identified longevity-related metabolites were associated with cancer risk, which indicated that some metabolic pathways linked to longevity in humans may be distinct from those involved in the development of morbid diseases [110].

Individuals with the same chronological age may exhibit different biological aging states and this individual variation can produce differences in the age-related clinical risk profiles and morbidity. By profiling the urine metabolites while using the NMR spectroscopy method, a measurement for biological age was constructed i.e., the metabolic age score, which is prognostic for weight loss in individuals who underwent bariatric surgery and it has applications in personalized medicine [117]. The lipid metabolism and lipoprotein particle size have been suggested to play a crucial role in longevity. Vaarhorst et al. reported that larger size low-density lipoprotein (LDL) particles and lower triglyceride (TG) levels are associated with long-lived families by performing a comprehensive lipid profiling analysis of the Leiden Longevity Study (LLS) group comprising of 421 families, and the sex-specific analysis revealed that LDL particle sizes were associated with male longevity, whereas TG levels (but not LDL particle size) associated with female longevity, thus suggesting a critical role of lipid metabolism in human longevity [114]. The plasma lipidome profiling analysis of 128 lipid species using the LC-MS approach revealed that 19 lipid species are associated with familial longevity in women with higher levels of ether phosphocholine (PC) and sphingomyelin (SM) species, and lower levels of phosphoethanolamine (PE) and long-chain triglycerides [113]. The longevity-associated lipid profile was also characterized by a higher ratio of the monounsaturated to polyunsaturated fatty acids, which suggested that the female plasma lipidome might be less prone to oxidative stress and harbor an efficient β-oxidation function [113].

The age-specific and sex-specific metabolic fingerprints were identified from the serum in 26,065 individuals of Northern European ancestry with significant differences being observed in the lipoproteins, cholesterol, and TG levels, with age in both genders, whereas the levels of atherogenic metabolites and certain amino acids were only found to increase in females during the time of menopausal transition [118]. A study by Jove et al. characterized the plasma metabolic profile of 150 healthy humans from 30 to 100 years of age, and identified the specific metabolites signature for each gender and also a set of gender-shared metabolites that included lipid species that significantly changed with age, indicating that altered lipid metabolism is closely linked to the aging process [109]. Individual metabolic differences in the human blood samples of 15 young and 15 elderly individuals that were analyzed by untargeted LC-MS approach revealed significant alterations in 14 compounds with age. The metabolites that declined with age included antioxidants and compounds that were involved in high physical activity (e.g., carnosine, UDP-acetyl-glucosamine, NAD^+^, and leucine), whereas metabolites that increased with age included those that were associated with a decline in renal and liver functions [59].

A recent study identified the aging-associated metabolites that are altered independently of chronological age in healthy individuals over time by performing the large population based longitudinal analyses from 590 KORA (Cooperative Research in the Region of Augsburg) healthy individuals and 386 healthy CARLA (Cardiovascular Disease, Living and Ageing in Haelle) participants using fasting serum samples by a targeted metabolomics approach [119]. This study identified several aging-associated altered metabolites, of which five metabolites (i.e., C18, arginine, ornithine, serine, and tyrosine) in women and four metabolites (i.e., arginine, ornithine, diacyl-phosphatidylcholine C36:3, and acyl-alkyl- phosphatidylcholine C40:5) in men were significantly altered with age, and arginine was decreased, whereas ornithine was increased with age in both sexes [119].

A cross-sectional KarMeN (Karlsruhe Metabolomics and Nutrition) study performed while using fasting blood and urine samples from 301 healthy men and women aged 18–80 years by targeted and untargeted metabolomics approaches revealed that sex and age can be predicted with high accuracy from the metabolite patterns of human plasma and urine [120]. The plasma metabolites levels of creatinine, branched-chain amino acids (i.e., leucine and isoleucine), uric acid, and sarcosine were higher in men, whereas that of creatine, phosphate, glycine, sphingomyelin (SM) C18:1, and phosphatidylcholines were higher in women, and the level of choline in plasma and level of sedoheptulose in urine enabled age prediction in both men and women, which suggested that sex and age exhibit a distinct metabolite signature in healthy humans [120].

Recently, the use of multiple analytical platforms allowed for a comprehensive, quantitative, and metabolome-wide characterization of human urine and plasma samples that lead to the identification of 445 unique urine metabolites and 4229 serum metabolites, respectively. A complete list with the identity and quantity of the detected metabolites, their structures, concentrations, and links to known disease associations are available in the urine metabolome database (UMDB: http://www.urinemetabolome.ca.) and serum metabolome database (SMDB: http://www.serummetabolome.ca) [23,25].

In summary, the omics technologies enable the quantitative analyses of biological molecules in a high-throughput manner at multiple levels, with high sensitivity and specificity. Although the field of metabolomics is relatively new when compared with other omics approaches (i.e., genomics, transcriptomics, and proteomics) in systems biology, it has emerged as a powerful tool for identifying global or systemic metabolite changes, discovering novel biomarkers, and understanding the complex networks in aging, and it thus holds a significant potential for enhancing our understanding of the biological mechanisms of aging in diverse model organisms. However, despite these significant advances, there are multiple challenges associated with omics studies in aging. First, the integration of high-throughput omics data is a major challenging problem in aging studies [37]. Second, the development of aging biomarkers also represents a significant challenge due to the inherent metabolic heterogeneity among individuals [121]. Third, the human cross-sectional studies generally tend to fail in capturing the real age-related phenomenon. Therefore, longitudinal measures from a large population cohort should be incorporated in aging studies to identify the novel biomarkers [122]. Fourth, making a clear distinction between the cause-and-consequence relationships among various altered factors in aging remains a challenging task. Developing new approaches that can integrate multi-level omics data is necessary for understanding the potential cause-and-consequence relationships and network interactions between multiple factors in aging.

It is notable that metabolites that are associated with amino acids, lipids, carbohydrate, TCA cycle, and redox metabolism may serve as biomarkers of aging and/or longevity, based on the metabolomics studies from model organisms and humans. However, how these diverse metabolites regulate the aging process and its complex networks remains unclear. In the future, high-throughput omics studies should be performed for delineating the unique and common mechanisms that underlie aging and lifespan extension in different organisms, and determining how different longevity associated genetic, diet, and environmental manipulations are related to each other. These studies could further determine which individuals may benefit the most from specific interventions based on the individual gene expression signatures or genetic background. Moreover, omics studies should be conducted at multiple time-points and with larger sample sizes to identify the bonafide biomarkers of aging and/or longevity.

## 3. Conclusions and Perspective

Aging is a natural biological phenomenon, albeit a complex trait that is influenced by individual genetics, diet, lifestyle, and environmental factors. In the past decade, the field of aging research has significantly accelerated with the advancements in high-throughput omics technologies. However, a complete understanding of the biological mechanisms of aging require the integration of omics data at multiple levels (i.e., transcriptomics, proteomics, and metabolomics), which is currently a major challenge, since it involves the integration of different types of data, including the annotation of pathways, network analysis, and interactions with environmental factors, such as diet and lifestyle.

The application of metabolomics to aging research is relatively new, and the use of MS based analytical platforms is becoming increasingly common due to its greater sensitivity and the ability to detect a large number of metabolites as compared with NMR, which is more useful for metabolic fingerprinting. Targeted and untargeted metabolomics approaches both display limitations; however, the ‘top-down’ untargeted metabolomics approach is relatively more useful in drawing conclusions in aging studies, since it captures a global snapshot of the organism’s metabolic state. In the past decade, a significant progress has been made in identifying global metabolites alterations that occur with age in humans. Thus, metabolomics is a highly useful and powerful tool in the studies of aging, since the metabolite analyses from human bio-fluids is minimally invasive and a cost-effective method for expediting clinical diagnosis with the goal to improve the health care of the aging population.

However, several key questions remain unanswered in the field of aging metabolomics. First, which age-dependent metabolic changes are the cause or consequence of aging? Second, how do complex molecular and regulatory networks change with age? Third, whether and how metabolites differences determine an organism’s lifespan? Fourth, which metabolites signatures associate with longevity? Fifth, how do metabolites patterns change in different species or organisms with diverse maximum lifespan? Sixth, is it possible to dissect and classify the cell-intrinsic, local, and systemic or cell-extrinsic metabolites that influence the aging process? As aging process exhibits heterogeneity between different organs and cell types within the same organ/tissue [123,124], it poses a significant challenge in mechanistically interpreting the omics data that were obtained from bulk tissues. Therefore, single-cell genome-wide analyses should be performed to infer the cell-to-cell heterogeneity in expression patterns during aging. Furthermore, to disentangle the potential cause-consequence relationships in aging, time-resolved longitudinal studies in vertebrates could be performed similarly to that described in yeast [125] for distinguishing the early occurring events that may be causative from later events that may emerge as a consequence.

Future studies that address or answer the abovementioned questions along with the development of new approaches that aid in efficiently integrating the multi-layered omics data would prove to be beneficial in enhancing our understanding of the biological mechanisms that regulate aging and longevity. Finally, the future of metabolomics studies lies in biomedical and clinical applications, including disease diagnosis, disease monitoring, preventive healthcare, and precision medicine, where it can be delivered to the masses and aid clinicians in making the best possible therapeutic and preventive interventions to improve patient care.

## Figures and Tables

**Table 1:** Metabolites changes in human biological fluids that are associated with aging and longevity.

Metabolites	Biofluids	Aging (↑↓)	Longevity (↑↓)	References
Arginine	Serum	↓	-	[119]
Ornithine, serine	Serum	↑	-	[119]
Creatinine, leucine, isoleucine, uric acid, sarcosine, phosphate, glycine, sphingomyelin (C18:1), phosphatidylcholines	Plasma	↑	-	[120]
Sedoheptulose	Urine	↓	-	[120]
Phosphoserine (40:5), monoacylglyceride (22:1), diacylglyceride (33:2), resolvin	Plasma	↓	-	[109]
25-hydroxy-hexacosanoic acid, eicosapentaenoic acid, phosphocholine (42:9), phosphoserine (42:3), 15-keto-prostaglandin F2α	Plasma	↓	-	[109]
l-γ-glutamyl-l-leucine	Plasma	↑	-	[109]
1,5-Anhydroglucitol, ophthalmic acid, carnosine, acetyl-carnosine, UDP-acetyl-glucosamine, NAD^+^, NADP^+^, leucine, isoleucine	Blood	↓	-	[59]
N_6_-acetyl-lysine, citrulline, pantothenate, dimethyl-guanosine, *N*-acetyl-arginine	Blood	↑	-	[59]
Lipoproteins	Serum	↑	-	[118]
Tryptophan	Serum	↓	-	[56,94]
C-glycosyl tryptophan,	Blood	↓	-	[111]
Creatine, β-hydroxy-β-methylbutyrate	Urine	↓	-	[112]
Acylcarnitines, diacyl phosphatidylcholines	Serum	↑	-	[56]
Amino acids	Serum	↓	-	[56]
Tricarboxylic acid intermediates	Plasma	↑	-	[115]
Creatine, urea, ornithine, polyamines	Plasma	↑	-	[115,120]
Essential, non-essential amino acids	Plasma	↑	-	[115]
Oxoproline, hippurate	Plasma	↑	-	[115]
Fatty acids, carnitine	Plasma	↑	-	[115]
Cholesterol, β-hydroxybutyrate	Plasma, serum	↑	-	[115,118]
Dehydroepiandrosterone-sulfate	Plasma	↓	-	[115]
Isocitrate, taurochlorate	Plasma	-	↓	[110]
Sphingomyelins	Serum	-	↓↑	[94,113]
Glycerophospholipids	Serum	-	↓↑	[94]
Phenylacetylglutamine, p-cresol sulfate	Urine	↑	↑	[94,112]
Ether phosphocholine, monounsaturated/polyunsaturated fatty acids ratio	Plasma	-	↑	[113]
Phosphoethanolamine	Plasma	-	↓	[113]
Low density lipoprotein size	Serum	-	↑	[114]
Triglycerides	Serum	↑	↓	[113,114,118]

Footnote: Metabolites that reportedly increased with aging and/or longevity in humans are marked with an upward arrow (↑) whereas those reported to decrease are marked with a downward arrow (↓).
